# L-Aspartic Acid Capped CdS Quantum Dots as a High Performance Fluorescence Assay for Sliver Ions (I) Detection

**DOI:** 10.3390/nano9081165

**Published:** 2019-08-14

**Authors:** Zhezhe Wang, Xuechun Xiao, Yue Yang, Tong Zou, Xinxin Xing, Rongjun Zhao, Zidong Wang, Yude Wang

**Affiliations:** 1 School of Materials Science and Engineering, Yunnan University, Kunming 650091, China; 2 Department of Physics, Yunnan University, Kunming 650091, China; 3 Key Lab of Quantum Information of Yunnan Province, Yunnan University, Kunming 650091, China

**Keywords:** L-Aspartic acid, CdS quantum dots, fluorescence assay, Ag^+^ detection, Fluorescence enhancement

## Abstract

A new high performance fluorescence assay for detection of Ag^+^ based on CdS quantum dots (QDs) using L-Aspartic acid (L-Asp) as a stabilizer was proposed in this work. The CdS quantum dots conjugation with L-Aspartic acid (L-Asp@CdS QDs) were successfully synthesized via a simple hydrothermal process. The QDs have a fluorescence emission band maximum at 595 nm with a quantum yield of 11%. The obtained CdS QDs exhibit a particle size of 1.63 ± 0.28 nm and look like quantum dot flowers. Basically, the fluorescence intensity of L-Asp@CdS QDs can be enhanced only upon addition of Ag^+^ and a redshift in the fluorescence spectrum was observed. Under optimum conditions, the fluorescence enhancement of L-Asp@CdS QDs appeared to exhibit a good linear relationship in between 100–7000 nM (R^2^ = 0.9945) with the Ag^+^ concentration, with a detection limit of 39 nM. The results indicated that the L-Asp@CdS QDs were well used in detection for Ag^+^ as fluorescence probe in aqueous solution with high sensitivity and selectivity. Moreover, the sensing system has been applied in detection Ag^+^ in real water samples. The recovery test results were 98.6%~113%, and relative standard deviation (n = 5) is less than 3.6%, which was satisfactory.

## 1. Introduction

Semiconductor quantum dots (QDs) [1,2], whose size is less than the material Bohr exciton radius, have received more and more attention due to their size dependence and novel optical properties during the past decades. Their three-dimensions confined to the nanoscales with a size typically in the range of 1–10 nm [1,3]. Unlike bulk materials, they have unique optical and electronic properties including wide excitation spectra, narrow symmetric and tunable emission spectra. Contemporarily, fluorescent semiconductor quantum dots, have attracted much attention in bio-imaging [4], solar cells [5], light-emitting diodes [6] and sensing [7,8]. Moreover, they have high photobleaching thresholds and excellent photostability advantages compared with traditional organic fluorescent dye.

CdS QDs is typical representatives of IIB-VIA semiconductor QDs (e.g., CdS, CdSe, CdTe and ZnS) [9]. However, the further applications of these semiconductor QDs in environmental detecting are limited by reason of their own toxicity. Therefore, organic molecules are selected as modifiers, which aim at reducing the surface defects and toxicity of semiconductor QDs. In recent years, surface functionalization and modification of QDs have widely applied as fluorescent probe in chemical and biological analysis [8,9,10,11,12]. So far, various organic ligands, such as thioglycolic acid [13], cysteamine [14] and 3-mercaptopropionic acid [8], citric acid [10] etc., have been applied in the quantum dots surface modification and functionalization. However, some modifiers are toxic, which do not comport with the green environmental protection science and technology philosophy. L-Aspartic acid (L-Asp), one of the essential amino acids in protein, is a naturally occurring amino acid without toxicity. Thus, CdS QDs surface-functionalized with L-Asp in water have been synthesized and characterized.

Currently, sliver is widely used in photographic materials, metallurgy, medicine, electroplating, etc. [15,16,17,18,19]. Trace amounts of Ag^+^ will harmless to people, but the U.S. Environmental Protection Agency reports that Ag^+^ could be harmful to fish and microorganisms when the concentration of Ag^+^ is higher than 1.6 nM [20]. According to the World Health Organization (WHO), the limitation of Ag^+^ in drinking water is 0.05 mg/L (467 nM) [21]. As a result, selective detection of Ag^+^ is important due to its severe pollution issue and potential toxicity. At present, the main method of monitoring the Ag^+^ mainly include inductively coupled plasma-mass spectrometry (ICP-MS), electrochemical and flame atomic absorption spectrometry (FAAS) [22,23,24]. However, those traditional methods have some shortcomings, such as poor selectivity, low sensitivity, time-consuming and tedious process. Recently, fluorescence probe is being focused gradually for the detection of free Ag^+^, which surmounts the weakness of other methods.

In this work, we studied fluorescent turn-on assay of Ag^+^ in aqueous solution based on L-Asp functionalized CdS QDs, which were synthesized by a facile hydrothermal method. It is found that the L-Asp@CdS QDs have a good selectivity to Ag^+^ ions from a coexisting solution system containing other metal ions, such as Al^3+^, Co^2+^, Cu^2+^, Fe^2+^, Fe^3+^, Hg^2+^, Na^+^, K^+^, La^3+^, Li^+^, Ni^2+^, Pb^2+^, Mn^2+^, Mg^2+^, Cd^2+^, Ca^2+^ and Zn^2+^. Besides, the present fluorescent sensor system has been applied to the Ag^+^ determination in real water samples and the results are agreeable.

## 2. Materials and Methods

### 2.1. Materials

Cadmium chloride hydrate (CdCl_2_·2.5H_2_O), thioacetamide (TAA), and sodium hydroxide (NaOH) were obtained from Aladdin Chemistry Co. Ltd. (Shanghai, China). Trihydroxymethylaminomethane (Tris), hydrochloric acid and L-Aspartic acid (L-Asp) were provided by Shanghai Macklin Biochemical Co. Ltd. (Shanghai, China). All metal salts were purchased from Tianjin Zhiyuan Chemical Reagent Co. Ltd. (Tianjin, China). The reagents were used without further purification.

### 2.2. Synthesis and Characterization of L-Asp@CdS QDs

L-Aspartic acid capped CdS QDs were synthesized in aqueous solution using a simple method. CdCl_2_·2.5H_2_O (0.195 g, 0.85 mmol) and L-Asp (0.2714 g, 2.04 mmol) were mixed with 50 mL ultrapure water in beaker with magnetic stirring. Five minutes later, the pH of the solution was adjusted to 10 with 4 mL of 1 M sodium hydroxide solution. Then, TAA (0.032 g, 0.425 mmol) was added and the mixture was stirred vigorously to homogeneity. Subsequently, the mixture was moved into a 100 mL steel autoclave and retained at 100 °C for 30 min. After being naturally cooled to room temperature, the obtained light yellow solution was removed to dialyze for 12 h with a 300 Da dialysis bag.

The morphological and structure of L-Asp@CdS QDs were analyzed by X-ray diffraction (XRD, Rigaku TTRIII-18KW, Rigaku Corporation, Tokyo, Japan) with a Cu K*α* radiation (*λ* = 1.54056 Å), transmission electron microscopy (TEM, 200 kV, JEOL, Tokyo, Japan), Renishaw inVia Raman microscope (Renishaw, London, UK), K-Alpha^+^ system X-ray photoelectron spectroscopy (XPS, Thermo Fisher Scientific Company, Waltham, MA, USA), AVATAR360 FT-IR spectrophotometer and Seiko SPA-400 SPM atomic force microscope (AFM, JEOL, Tokyo, Japan). The optical properties of L-Asp@CdS QDs were acquired on the Horiba Fluorolog-3 spectrofluorometer (Horiba, NY, USA) and Jinghua Instruments UV-1800 spectrophotometer (Quantaurus-Tau, Hamamatsu, Japan). The pH of the current system was monitored by an inoLab pH Level 1 precision pH meter (Weilheim, Germany). The fluorescence quantum yield was computed by the following equation [10]:

QY_(sample)_ = (*F*_sample_/*F*_ref_) (*A*_ref_/*A*_sample_) (*η*_sample_^2^/*η*_ref_^2^) QY_ref_ (1)

Rhodamine 6G was selected as the reference standard and its solvent was ethanol (QY = 95%). *F*, *A* and *η* are the area under the emission spectra, the absorbance at the excitation wavelength and the refractive index of solvents, respectively.

### 2.3. Detection of Ag^+^ by L-Asp@CdS QDs

Fluorescence enhancement of the L-Asp@CdS QDs by Ag^+^ was carried out with a typical procedure. An amount of 250 μL L-Asp@CdS QDs solution and 100 μL various concentrations of Ag^+^ were diluted with 4 mL of distilled water in 10 mL plastic centrifuge tube. Next, the plastic centrifuge tube was filled to 5 mL with 0.3 M Tris–HCl buffered solution. The L-Asp@CdS QDs solution was diluted to 5 mL with ultrapure water. Finally, Ag^+^ concentration was obtained on the range from 100 nM to 10 µM. Relative fluorescence intensity (F/F_0_, F_0_ and F are the fluorescence intensity of L-Asp@CdS QDs before and after addition of metal ions, respectively) was used to represent the fluorescence enhancement efficiency. These solutions were analyzed at *λ*_em_/*λ*_ex_ = 595/405 nm. To verify the feasibility of the fluorescence assay, drinking water from the Yunnan spring, Kunming City, was as the real water sample.

### 2.4. Interference Studies

The following procedure was demonstrated to study the selectivity of L-Asp@CdS QDs to Ag^+^. All metal salts were purchased from Kunming Maidesen: KCl, NaCl, CdCl_2_·2.5H_2_O, BaCl_2_·2H_2_O, CaCl_2_, FeCl_3_·6H_2_O, CuCl_2_·2H_2_O, FeCl_2_·4H_2_O, AlCl_3_·6H_2_O, MnCl_2_·4H_2_O, NiCl_2_·6H_2_O, CoCl_2_·6H_2_O, ZnCl_2_, Mg(NO_3_)_2_·6H_2_O, Pb(NO_3_)_2_, LaCl_3_·7H_2_O, HgCl_2_ and LiNO_3_. The stock solution of all metal ions (2.5 mM) was prepared in ultrapure water, which were diluted to various concentrations were used in experimental testing. An amount of 250 μL L-Asp@CdS QDs solution and 100 μL a certain concentration interfering metal ions were diluted with 4 mL of distilled water in 10 mL plastic centrifuge tube. Next, the plastic centrifuge tube was filled to 5 mL with 0.3 M Tris–HCl buffered solution. Then the concentrations of all interfering metal ions was 50 μM.

## 3. Results and Discussion

### 3.1. Characterization of the L-Asp@CdS QDs

**Figure 1:**
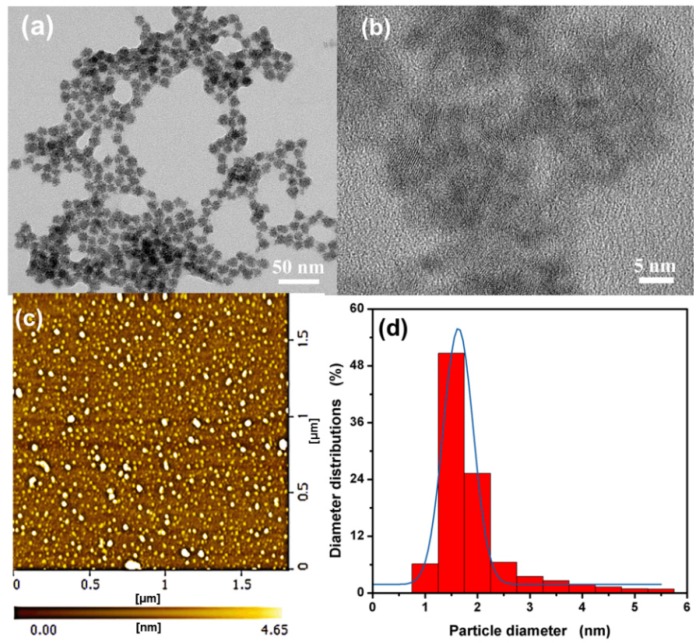
Transmission electron microscopy (TEM) (**a**), high-resolution TEM (HRTEM) (**b**,**c**) atomic force microscope (AFM) images of as-synthesized L-Aspartic acid (L-Asp)@CdS QDs, (**d**) statistical analysis of the heights of L-Asp@CdS QDs measured by AFM.

Figure 1 shows the morphology and particle size of the as-synthesized L-Asp@CdS QDs. The TEM image (Figure 1a) and high-resolution TEM (HRTEM) image (Figure 1b) illustrate that the uniform quantum dot flowers (about 10 nm) are composed of L-Asp@CdS QDs smaller than 2 nm. As can be observed from the AFM images (Figure 1c) and the height distribution histograms (Figure 1d) of L-Asp@CdS QDs, the surface of sample is very uniform, with height of 1.63 ± 0.28 nm, which is consistent with the result of the TEM.

**Figure 2:**
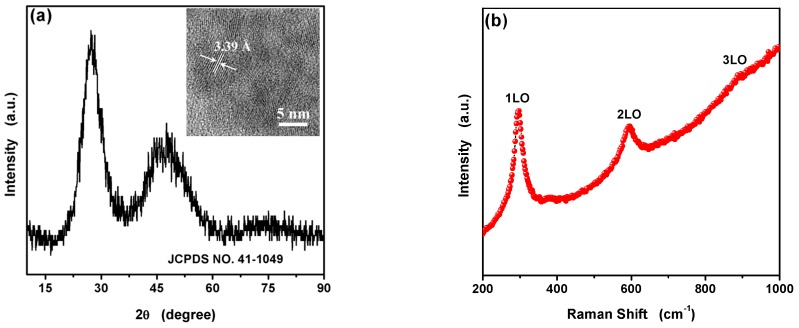
X-ray diffraction pattern (**a**) and Raman spectra (**b**) of as-synthesized L-Asp@CdS quantum dots (QDs). The inset of (**a**) shows the corresponding HRTEM image of L-Asp@CdS QDs.

XRD and Raman were used to analyze the compositions and crystalline structures of the obtained product. The XRD patterns (Figure 2a) indicate that the peaks at 2θ = 27.3°, 45.1° and 48.2° correspond to (002), (110) and (103) plane of hexagonal wurtzite structure (JCPDS No. 41-1049) of CdS, respectively. The HRTEM image of the L-Asp@CdS QDs (Figure 2a, inset) shows the crystalline interplanar spacing was 3.38 Å, which aligns with the (002) plane. The Raman spectra further proved the synthesis of CdS. As can be observed in Figure 2b, the Raman modes (296 cm^−1^, 596 cm^−1^, 893 cm^−1^) are agreement with CdS, which are lower than the reported values of bulk CdS [25,26]. This could be construed as the dipole–dipole interactions between L-Asp@CdS QDs caused many normal modes resulting in broadening and shifting of surface phonon peaks due to the L-Asp@CdS QDs aggregations [26].

**Figure 3:**
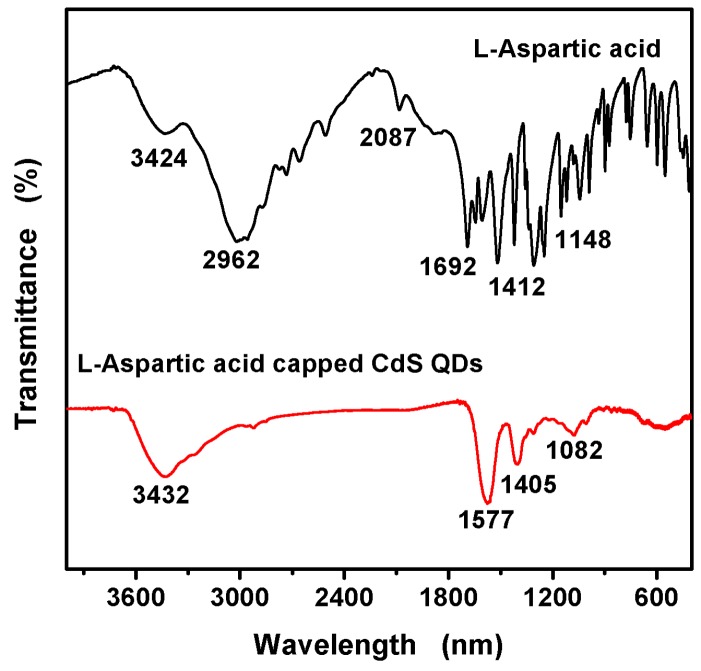
FTIR spectra of L-Asp (black line) and L-Asp@CdS QDs (red line).

The IR spectra of pure L-Asp and L-Asp@CdS QDs are displayed in Figure 3. The characteristics groups of L-Asp are –COOH and –NH_2_. For L-Asp, its spectrum shown the peaks at around 3424 cm^−1^, 2962 cm^−1^, 2087 cm^−1^, 1692 cm^−1^, 1412 cm^−1^ and 1148 cm^−1^ are assigned to the N–H (sν_N-H_), O–H (sν_O-H_), C–H (sν_C-H_), C=O (sν_C=O_), COO– (mν_COO__H_), and C–NH_2_ (mν_C-N_) [27,28,29]. For the FTIR spectrum of the L-Asp-CdS QDs, the feature band of O–H (sν_O-H_) disappears, the band of C=O (sν_C=O_) shifts from 1692 to 1577 cm^−1^ and the band of C–NH_2_ (mν_C-N_) shifts from 1148 to 1082 cm^−1^. As a result, L-Asp successfully decorated on the surface of CdS QDs, which are bonded by carboxyl groups rather than amino group.

**Figure 4:**
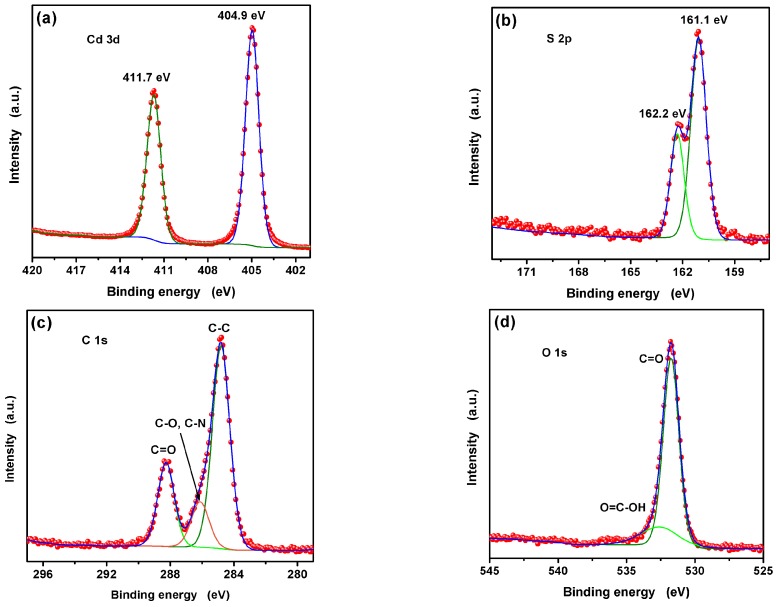
X-ray photoelectron spectroscopy (XPS) spectra of the synthesized L-Asp@CdS QDs: (**a**) Cd 3d spectrum, (**b**) S 2p spectrum, (**c**) C 1s and (**d**) O 1s spectrum, respectively.

To further verify the surface element composition and chemical state, the XPS spectra of L-Asp@CdS QDs was researched as indicated in Figure 4. The Cd and S spectra are displayed in Figure 4a,b, respectively. The Cd 3d peaks are observed at 411.7 eV and 404.9 eV, which can be assigned to Cd 3d_3/2_ and Cd 3d_5/2_. For the peaks of S, located at 161 eV and 162.2 eV, belong to S 2p_3/2_ and S 2p_1/2_. The +2 oxidation state of Cd and −2 oxidation state of S confirm the existent of CdS [30,31]. As displayed in Figure 4c, the C 1s peaks appeared at 284.8 eV, 286.1 eV and 288.2 eV was attribute to the C–C, C–O/C–N and C=O, respectively. The O 1s XPS spectra (Figure 4d) revealed two peaks located at 531.7 and 532.8 eV which correspond to C=O and O=C–OH, respectively [32]. The presence of carboxyl groups suggests that L-Asp is not a polydentate ligand. Distinctly, the two carboxylate groups of L-Asp are not attached to the surface with equal strength because one of them relates to zwitterion process and can be better connected with CdS QDs [32,33]. Thus, the results proved that the L-Asp@CdS QDs have been successfully synthesized.

### 3.2. Optical Properties of Synthesized L-Asp@CdS QDs

**Figure 5:**
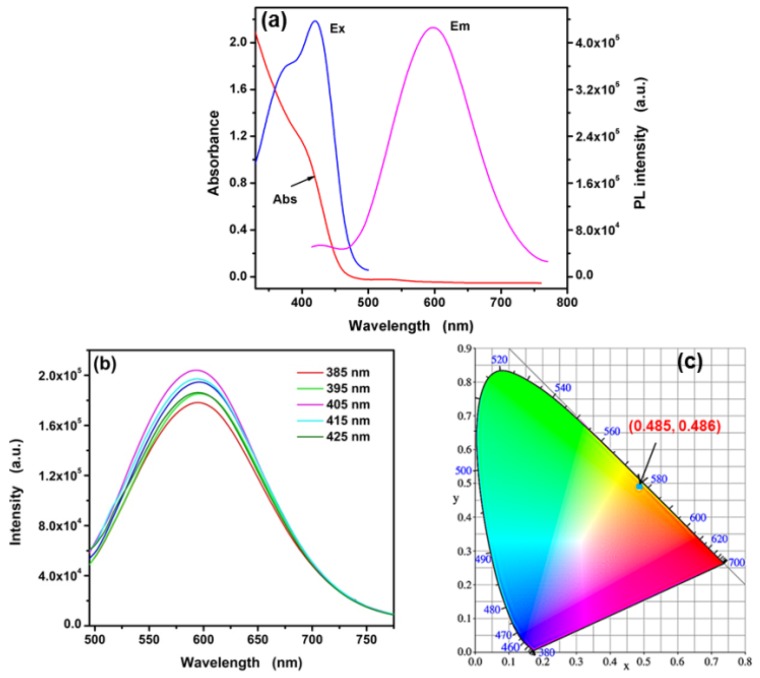
(**a**) UV-visible absorption (red line), emission spectrum (pink line), excitation spectrum (blue line), and (**b**) excitation-related emission spectra of L-Asp@CdS QDs, and (**c**) CIE 1931 coordinates.

The UV-vis absorption spectrum and fluorescence spectra were carried for studying the optical properties of L-Asp@CdS QDs. As shown in Figure 5a, the broad absorption band with an absorption edge (the sharply decreasing region of the UV–Vis spectrum of intersecting with the baseline) about 459 nm is obtained. The optical bandgap is estimated to be 2.7 eV, which has taken place blue shift contrast with bulk CdS (2.42 eV) due to the quantum confinement of L-Asp@CdS QDs [10]. The maximum emission intensity is observed at 595 nm and there are two excitation peaks, which are 419 nm and 376 nm, respectively. The excitation-dependent emission spectra (Figure 5b) show that the emission peak basic remains unchanged under the regulation of excitation wavelength from 385 nm to 425 nm. When the excitation wavelength is 405 nm, the maximum emission intensity of L-Asp@CdS QDs is obtained. As shown in Figure 5c, CIE coordinates of L-Asp@CdS QDs is located at (0.485, 0.486) with a correlated color temperature of 2913 K. The L-Asp@CdS QDs emit orange-yellow light under excitation wavelength of 405 nm. The quantum yield (QY) of the synthetic L-Asp-CdS QDs was calculated to be 9.24% using rhodamine 6G as a reference, the low fluorescence quantum yields could be due to the aggregation of L-Asp-CdS QDs.

### 3.3. Ag^+^ Detection Based on the L-Asp@CdS QDs

In this work, the detection of silver is based on L-Asp@CdS quantum dot fluorescence enhancement. The affecting factors of pH and concentration were studied in order to obtain the optimal properties to detection Ag^+^ based on the L-Asp@CdS QDs. The results released in Figure S1. As can be discovered, the emission intensity of L-Asp@CdS QDs exhibits the maximum at pH 9.0 with a maximum fluorescence enhancement. Moreover, the maximum emission intensity was obtained when 0.5 mL L-Asp@CdS QDs was be diluted to 5 mL simultaneously. It exhibits great relative fluorescence intensity (F/F_0_) before and after addition of Ag^+^. Therefore, pH 9.0 and 1.5 mL L-Asp@CdS QDs was chosen as the optimal condition and applied in the next experiments.

**Figure 6:**
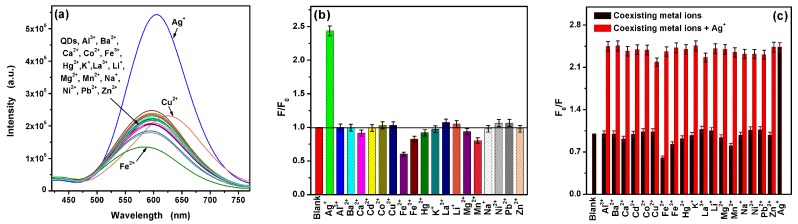
(**a**) Fluorescence spectra of L-Asp@CdS QDs in the presence of various cations (**b**) plots of relative fluorescence intensity (F_0_/F) in the presence of various cations and (**c**) selective fluorescence response of L-Asp@CdS QDs to 10 μM Ag^+^ (wine bars), and interference cations.

**Figure 7:**
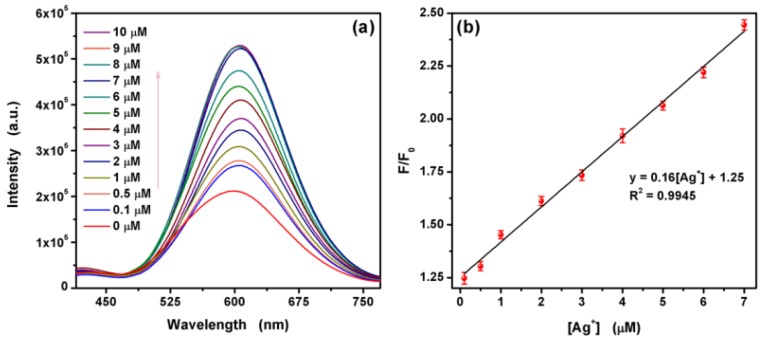
(**a**) Fluorescence spectra of synthesized L-Asp@CdS QDs solution upon addition of various concentrations of Ag^+^, (**b**) relative fluorescence intensity of L-Asp@CdS QDs in the presence of various concentrations of Ag^+^ (the error bar indicates the standard deviation, which was obtained by repeated the experiments 5 times).

The sensitivity of L-Asp@CdS QDs was investigated based on the change of fluorescence intensity. The fluorescence spectra upon addition of various metal ions are shown in Figure 6a. It is observed that a strong enhancement of fluorescence intensity is observed with the addition of Ag^+^. Meanwhile, the emission spectra exhibit a red shift. Figure 6b depicts the relative fluorescence intensity. As can be seen, there are no obvious fluorescence enhancement when add other metal ions including Al^3+^, Co^2+^, Cu^2+^, Fe^2+^, Fe^3+^, Hg^2+^, Na^+^, K^+^, La^3+^, Li^+^, Ni^2+^, Pb^2+^, Mn^2+^, Mg^2+^, Cd^2+^, Ca^2+^ and Zn^2+^. To evaluate the resistant to interference of L-Asp@CdS QDs for Ag^+^ detection, the mixtures of 10 μM Ag^+^ and 50 μM coexisting metal ions were added into L-Asp@CdS QDs solution respectively, then the fluorescence enhancement were carried out. Figure 6c represents that the impact of interfering metal ions is paltry on the fluorescence enhancement. Therefore, the present fluorescence assay system has highly selective and outstanding anti-interference for Ag^+^ detection. Figure 7a shows a positive correlation between the fluorescence intensity of L-Asp@CdS QDs and the concentration of Ag^+^. The significant and gradual enhance of the fluorescence intensity is noticed upon addition the varied concentration of Ag^+^ from 100 nM to 10 μM. As a result, the fluorescence assay system based on L-Asp@CdS QDs is sensitive to the Ag^+^. Figure 7b indicates a good linear relationship between the relative fluorescence intensity and the concentration of Ag^+^ from 100 nM to 7 μM. The fitting liner equation is F/F_0_ = 0.16[Ag^+^] + 1.25 with a fantastic correlation coefficient (R^2^) of 0.9945. On the basis of this data, the detection limit (LOD) for Ag^+^ was obtained to be 39 nm based on a signal-to-noise ratio (S/N) of 3, which is lower than the limit content in drinking water published by the WHO. The performance comparison of Ag^+^ detection based on several fluorescence assays have been listed in Table 1. As can be seen, the detection limit of the present assay was relatively low with a satisfactory sensitivity.

### 3.4. Ag^+^ Detection on Real Water Sample

To evaluate the practicability of the synthesized L-Asp@CdS QDs for Ag^+^ detection, the fluorescence assay was investigated in real drinking water. The water samples are added with Ag^+^ to be the certain concentration of 0.1, 0.5 and 1.0 μM. The results are shown in Table 2, which indicate that the measured concentrations are good agreement with the spiked value. The recoveries of Ag^+^ in real water sample are in the range of 98.6–112%. Moreover, the relative standard deviations (RSD) are below 5% and illustrate that the fluorescence assay has high accuracy. As a result, the fluorescence assay based on the L-Asp@CdS QDs presents excellent sensitivity and has an enormous potential in environmental applications.

### 3.5. Mechanism of Ag^+^ Detection Based on the L-Asp@CdS QDs

**Figure 8:**
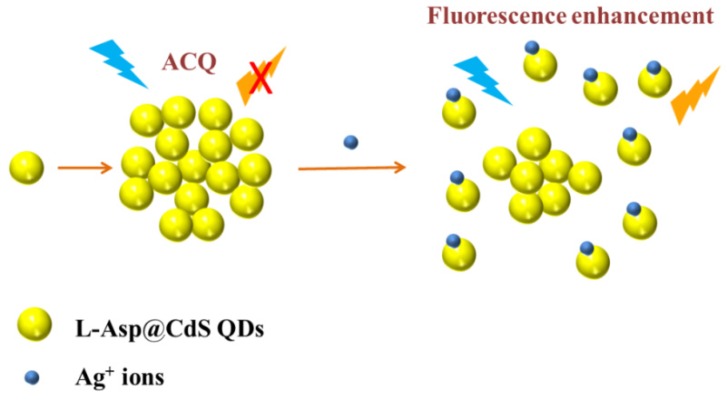
Schematic diagram for L-Asp@CdS QDs fluorescence enhancement by Ag^+^ ions.

In order to explore the fluorescence enhancement mechanism of L-Asp@CdS QDs by Ag^+^, the TEM image, XRD, XPS and UV-vis spectra of L-Asp@CdS QDs after adding Ag^+^ were carried out. From Figure S2 it can be seen that no new phase can be formed. Therefore, the presence of silver sulfide (Ag_2_S) is excluded. As displayed in Figure S3, there was no significant new absorption peak appeared upon addition of Ag^+^ ions, which further evidence that Ag^+^ ions do not react with L-Asp@CdS QDs to form Ag_2_S. The XPS information is confirmed the Ag^+^ ions are adsorbed onto the L-Asp@CdS QDs surface. Here, the spectra of survey, Cd 3d, S 2p, Ag 3d, C 1s and O 1s are shown in Figure S4. The appearance of Ag 3d peaks at 373.7 eV and 367.7 eV respectively corresponding to Ag 3d_3/2_ and Ag 3d_5/2_ [34], which suggest the Ag^+^ ions is adsorbed onto the L-Asp@CdS QDs surface. As shown in Figure S5, the TEM image of after addition of Ag^+^ ions shows a better dispersion compared with before the addition of Ag^+^ ions. Therefore, the mechanism of fluorescence enhancement by Ag^+^ can be speculated and expressed at Figure 8. The fluorescence of the L-Asp@CdS quantum dots in the aggregation state is weak, which is attributed to the aggregation caused quenching (ACQ) effect [40]. This is owing to the intermolecular π-π/n-π * interactions or other non-radiative channels, which in turn quench its emission [41]. After addition of Ag^+^, Ag^+^ adsorbed on the L-Asp@CdS QDs surface and generated electrostatic repulsion. Thus, the L-Asp@CdS QDs dispersion was improved and the ACQ effects were weakened. As a result, Ag^+^ ions adsorbing on the surface of L-Asp@CdS QDs can reduce the non-radiative electron/hole recombination process and then result in fluorescence enhancement.

## 4. Conclusions

In summary, the hydrothermal method has been successfully first used in the synthesis of L-aspartic acid stabilized CdS quantum dots. The synthesized L-Asp@CdS QDs show an outstanding selectivity and sensitivity for Ag^+^ ions detection by fluorescence enhancement. As a result, an excellent linear relationship was found in the range of 0.1–7 μM with LOD of 0.039 μM, which is below the minimum level of Ag^+^ ions in drinking water published by the WHO. Moreover, the proposed fluorescence assay has been applied in the real drinking water and a pretty result was obtained. Hence, the present fluorescence assay has a potential application for Ag^+^ detection in environment water sample.

## Figures and Tables

**Table 1:** Comparison the analytical performance of Ag^+^ detection based on fluorescence assay.

Fluorescent Probe	Detection Mode	Linear Range (μM)	Detection Limit (μM)	Sensitivity (μM^−1^)	Ref.
CdTe QDs	^a^ F.Q.	2–10	0.1	Not given	[34]
GQDs	Ratiometric	0–115.2	0.25	0.01649	[35]
S-GQDs	^a^ F.Q.	0.1–130	0.03	0.0053	[36]
GSH@CdTe QDs	^b^ F.E.	0.02–0.2	0.0013	0.4626	[37]
TLA-CdTe QDs	^a^ F.Q.	0.05–10/10–100	0.05	0.1151/0.055	[38]
Cys-CdS QDs	^b^ F.E.	0.1–1.5	0.068	74.71	[39]
L-Asp@CdS QDs	^b^ F.E.	0.1–7	0.039	0.16	This work

^a^ F.Q. Fluorescence quenching, ^b^ F.E. Fluorescence enhancement.

**Table 2:** Analytical results of drinking water samples.

Samples	Spiked (μM)	Found (μM)	Recovery (%, n = 5)	Relative Standard Deviation (RSD) (%, n = 5)
Drinking water	0.500	0.493	98.6	3.6
	1.000	1.121	112	2.1
	3.000	3.168	105	2.4

## Supplementary Materials

The following are available online at https://www.mdpi.com/2079-4991/9/8/1165/s1, Figure S1: (a) Fluorescence spectra at different volumes of L-Asp@CdS QDs in buffer solution at pH 9 and (b) luorescence intensity without (red) and with (violet) 10 µM Ag^+^, and the relative fluorescence intensity of volume dependence (blue) of L-Asp@CdS QDs, (c) effect of pH on the fluorescence intensity and (d) fluorescence intensity without (red) and with (violet) 10 µM Ag^+^, and relative fluorescence intensity of pH dependence (blue)of L-Asp@CdS QDs, Figure S2: XRD patterns of L-Asp@CdS QDs in the absence and presence of Ag^+^ ions, Figure S3: UV–vis absorption spectra of L-Asp@CdS QDs in the presence of various concentrations of Ag^+^ ions, inset, the photographs under exposure of white and UV (365 nm) light, Figure S4: XPS spectra of the L-Asp@CdS QDs upon addition of Ag^+^ ions: (a) survey, (b) Cd 3d spectrum, (c) S 2p spectrum, (d) Ag 3d, (e) C 1s and (f) O 1s spectrum, Figure S5: The TEM and HRTEM images of L-Asp@CdS QDs upon addition of Ag^+^ ions, Table S1: The information of Yunnan Spring.
