# Supplementary material for: L-Aspartic Acid Capped CdS Quantum Dots as a High Performance Fluorescence Assay for Sliver Ions (I) Detection
Source: Nanomaterials (Basel). 2019 Aug 14;9(8):1165. doi: 10.3390/nano9081165 (PMC6724099; doi:10.3390/nano9081165)
Supplement: Supplementary file 1 [file nanomaterials-09-01165-s001.pdf]

Supporting Information

L-Aspartic Acid Capped CdS Quantum Dots as a High Performance Fluorescence Assay for Sliver Ions (I) Detection

Zhezhe Wang [1,2], Xuechun Xiao [2,*], Yue Yang [2], Tong Zou [1], Xinxin Xing [2], Rongjun Zhao [1,2], Zidong Wang [1] and Yude Wang [1,3,*]

1 School of Materials Science and Engineering, Yunnan University, Kunming 650091, China
2 Department of Physics, Yunnan University, Kunming 650091, China
3 Key Lab of Quantum Information of Yunnan Province, Yunnan University, Kunming 650091, China
* Correspondence: xchxiao@ynu.edu.cn (X.X.); ydwang@ynu.edu.cn (Y.W.); Tel: +86-871-65930112 (X.X.) Tel.: +86-871-65035570 (Y.W.); Fax: +86-871-65153832 (Y.W.)

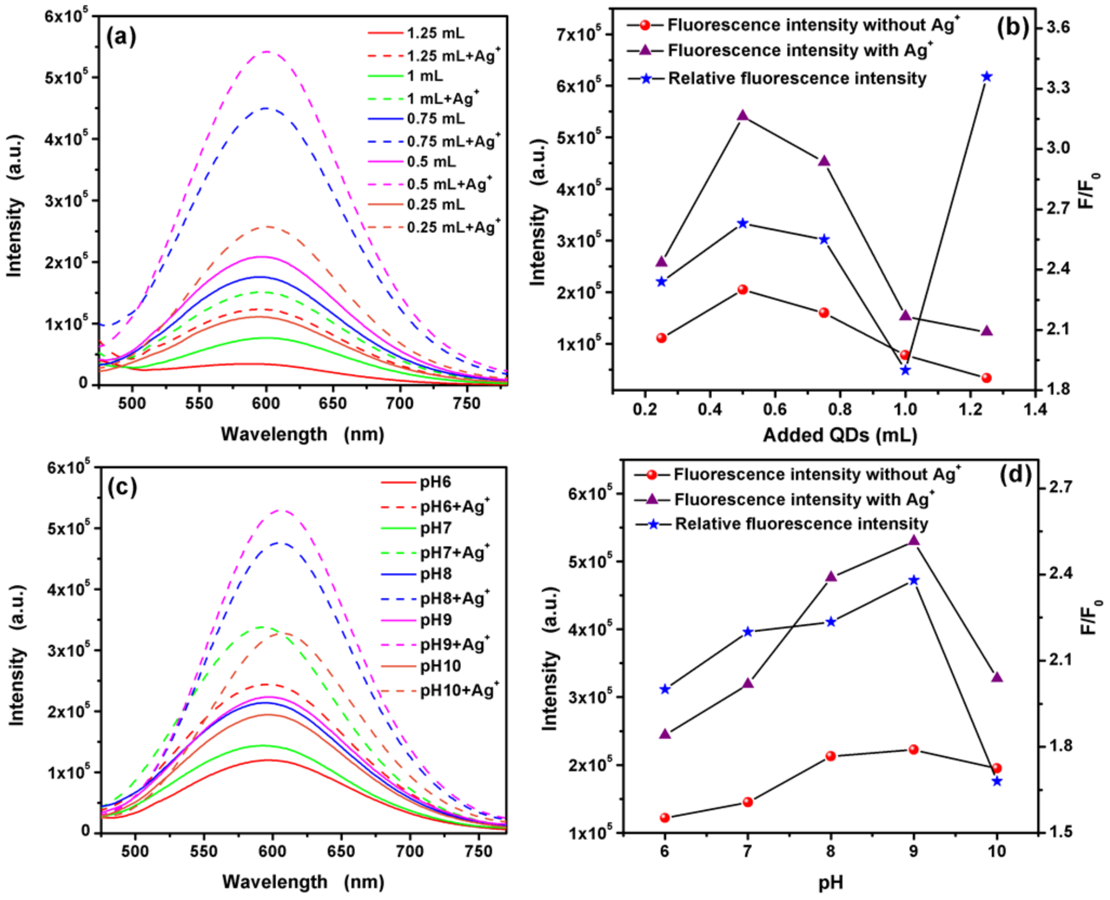

Figure S1. (a) Fluorescence spectra at different volumes of L-Asp@CdS QDs in buffer solution at pH 9 and (b) luorescence intensity without (red) and with (violet) 10 μM Ag^+, and the relative fluorescence intensity of volume dependence (blue) of L-Asp@CdS QDs, (c) effect of pH on the fluorescence intensity and (d) fluorescence intensity without (red) and with (violet) 10 μM Ag^+, and relative fluorescence intensity of pH dependence (blue)of L-Asp@CdS QDs.

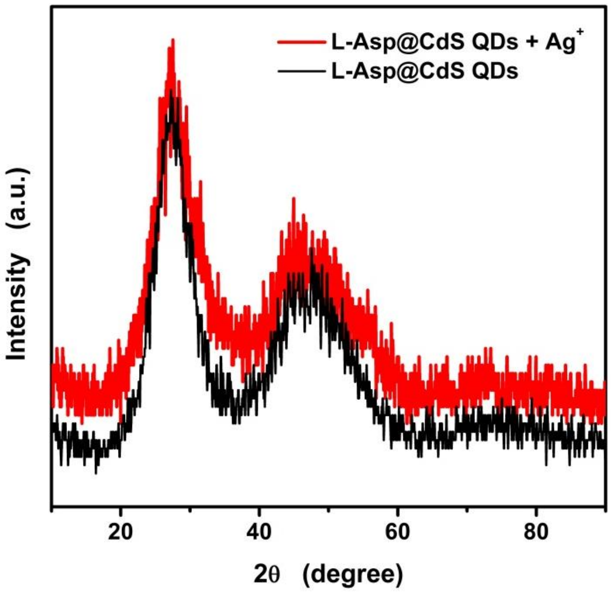

Figure S2. XRD patterns of L-Asp@CdS QDs in the absence and presence of Ag^{+} ions.

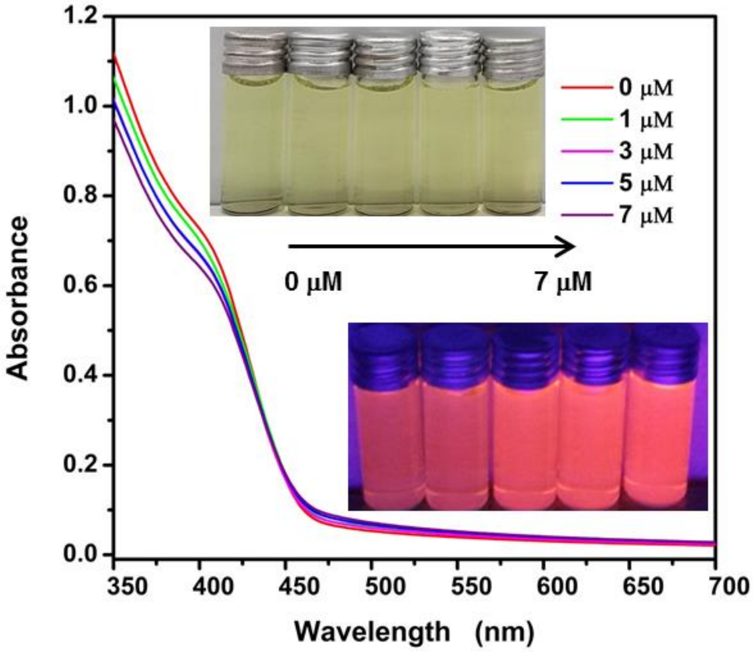

Figure S3. UV–vis absorption spectra of L-Asp@CdS QDs in the presence of various concentrations of Ag+ ions, inset, the photographs under exposure of white and UV (365 nm) light.

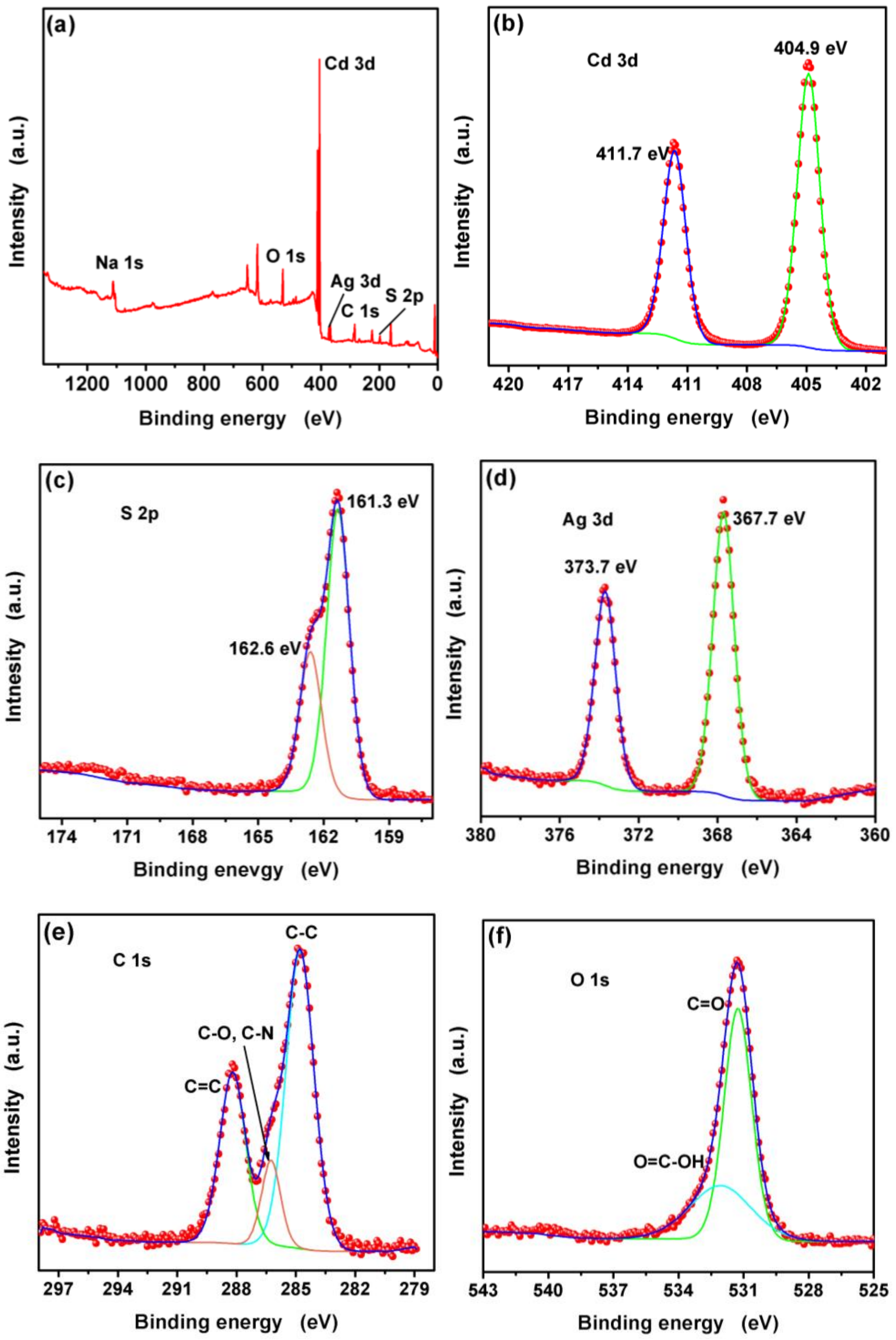

Figure S4. XPS spectra of the L-Asp@CdS QDs upon addition of Ag+ ions: (a) survey, (b) Cd 3d spectrum, (c) S 2p spectrum , (d) Ag 3d, (e) C 1s and (f) O 1s spectrum.

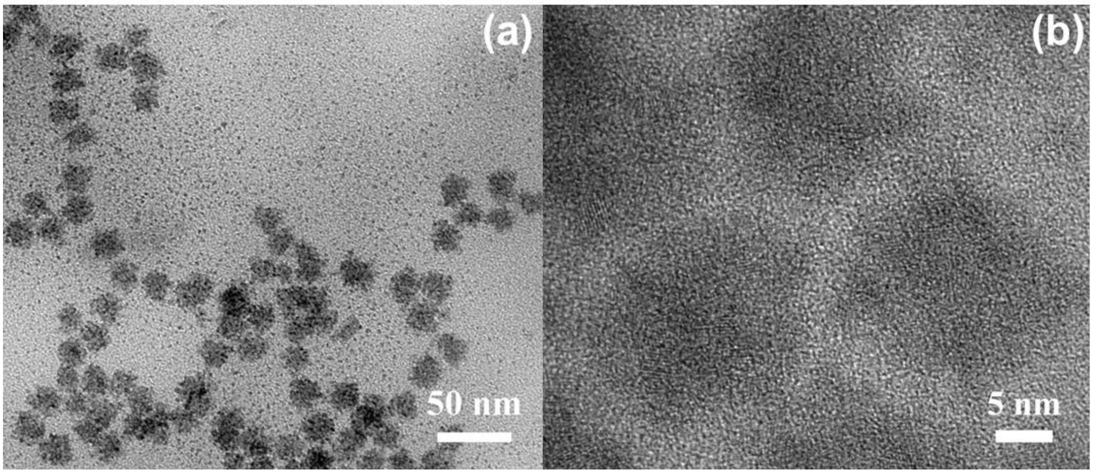

Figure S5. The TEM and HRTEM images of L-Asp@CdS QDs upon addition of Ag^{+} ions.

Table S1. The information of Yunnan Spring.

Mineral composition (mg/L)		Total dissolved solids (mg/L)	pH
H_2SiO_3	20.0–60.0	45.0–150.0	6.5–8.5
Ca^{2+}	3.0–15.0		
Mg^{2+}	0.03–5.0		
